# Comparative Genomic Analysis of Clinical Strains of *Campylobacter jejuni* from South Africa

**DOI:** 10.1371/journal.pone.0002015

**Published:** 2008-04-23

**Authors:** Beatriz Quiñones, Magalie R. Guilhabert, William G. Miller, Robert E. Mandrell, Albert J. Lastovica, Craig T. Parker

**Affiliations:** 1 United States Department of Agriculture-Agricultural Research Service, Produce Safety and Microbiology Research Unit, Albany, California, United States of America; 2 AgraQuest, Inc., Davis, California, United States of America; 3 Department of Biotechnology, University of the Western Cape, Bellville, South Africa; National AIDS Research Institute, India

## Abstract

**Background:**

*Campylobacter jejuni* is a common cause of acute gastroenteritis and is also associated with the post-infectious neuropathies, Guillain-Barré and Miller Fisher syndromes. In the Cape Town area of South Africa, *C. jejuni* strains with Penner heat-stable (HS) serotype HS∶41 have been observed to be overrepresented among cases of Guillain-Barré syndrome. The present study examined the genetic content of a collection of 32 South African *C. jejuni* strains with different serotypes, including 13 HS∶41 strains, that were recovered from patients with enteritis, Guillain-Barré or Miller Fisher syndromes. The sequence-based typing methods, multilocus sequence typing and DNA microarrays, were employed to potentially identify distinguishing features within the genomes of these *C. jejuni* strains with various disease outcomes.

**Methodology/Principal Findings:**

Comparative genomic analyses demonstrated that the HS∶41 South African strains were clearly distinct from the other South African strains. Further DNA microarray analysis demonstrated that the HS∶41 strains from South African patients with the Guillain-Barré syndrome or enteritis were highly similar in gene content. Interestingly, the South African HS∶41 strains were distinct in gene content when compared to HS∶41 strains from other geographical locations due to the presence of genomic islands, referred to as *Campylobacter jejuni* integrated elements (CJIEs). Only the integrated element CJIE1, a *Campylobacter* Mu-like prophage, was present in the South African HS∶41 strains whereas this element was absent in two closely-related HS∶41 strains from Mexico. A more distantly-related HS∶41 strain from Canada possessed both integrated elements CJIE1 and CJIE2.

**Conclusion/Significance:**

These findings demonstrate that CJIEs may contribute to the differentiation of closely-related *C. jejuni* strains. In addition, the presence of bacteriophage-related genes in CJIE1 may contribute to the genomic diversity of *C. jejuni* strains. This comparative genomic analysis of *C. jejuni* provides fundamental information that potentially could lead to improved methods for analyzing the epidemiology of disease outbreaks.

## Introduction

Illnesses caused by bacterial foodborne pathogens continue to be a serious health issue worldwide. In laboratory-confirmed cases of infection, *Campylobacter jejuni* is considered to be one of the most significant bacterial causes of human gastroenteritis [1]–[3]. The majority of *C. jejuni* infections result in an acute, self-limited gastrointestinal illness. However, in a small number of patients, *C. jejuni* infection is followed by complications, including septicaemia [4] and the development of the autoimmune neuropathies, Guillain-Barré (GBS) and Miller-Fisher (MFS) syndromes [5]–[7]. The development of these autoimmune neuropathies after *C. jejuni* infection is thought to be related to sialylated lipooligosaccharides (LOS) on the cell surface of *C. jejuni* that exhibit molecular mimicry with gangliosides on peripheral nerves [8]–[11]. The range of *C. jejuni* disease outcomes, extending from acute inflammatory diarrhea to the induction of the autoimmune neuropathies, could be associated with the differential expression of virulence factors in *C. jejuni* strains. Additionally, the broad variability in the severity and spectrum of clinical symptoms in GBS patients suggests that host susceptibility may play a substantial role in disease development [12], [13].

Recent studies have characterized collections of strains from patients with GBS in search of a specific *C. jejuni* genotype responsible for the development of this neuropathy. *C. jejuni* strains from GBS cases, with Penner heat-stable (HS) serotypes HS∶19 and HS∶41, have been shown to be overrepresented in Japan and South Africa, respectively [14]–[17]. Further genetic characterization of GBS-associated strains with HS∶41 and HS∶19 demonstrated that these serotypes represent clonal populations [18], [19], suggesting that specific virulence traits relevant to the onset of GBS may be present in strains with these serotypes. Although strains with other common serotypes have been isolated from patients with GBS, the clustering of GBS-associated strains into specific serotypes has not been observed to occur in all worldwide geographical locations [20], [21].

A major contributor to the development of GBS that is distinct from capsular polysaccharide, the Penner serotyping determinant [22], is thought to be *C. jejuni* LOS. In most patients who develop GBS after *C. jejuni* enteritis, an induction of their immune response occurs possibly due to anti-*C. jejuni* LOS antibodies that cross-react with ganglioside epitopes on neural tissues [7]. In particular, LOS locus classes A, B, and C contain genes involved in the biosynthesis and transfer of sialic acid, an essential component of gangliosides [23], [24], and certain genes in the biosynthesis of these specific LOS classes are proposed to be associated with the induction of the immune response [8], [10], [11], [25]. Further analyses demonstrated that a high frequency of GBS-associated strains possessed LOS locus class A [8], [9], [26], [27] while MFS-associated strains had LOS locus class B [8], [26]. Potentially, a comparative genomic study of a highly-similar collection of *C. jejuni* strains associated with various disease outcomes could lead to the discovery of those determinants that may contribute to the development of GBS.

Various sequence-based typing methods, including multilocus sequence typing (MLST) [28]–[34], DNA sequencing of certain virulence loci [24], [27], [35] and comparative gene indexing by DNA microarrays [36]–[42], have been exploited to both identify differences between *C. jejuni* strains and potential relationships between molecular markers and disease outcomes. In particular, gene indexing studies, by using whole-genome microarrays of *C. jejuni* strain NCTC 11168, have demonstrated several regions of intraspecies genome hypervariability between strains of *C. jejuni*, including the LOS, capsular polysaccharide, flagellar biosynthetic and restriction-modification loci [36]–[42].

The genome sequence data of *C. jejuni* strain RM1221 have provided additional information of intraspecies genome diversity in *C. jejuni* [43]. The genome of strain RM1221 is syntenic with the genome of *C. jejuni* NCTC 11168 except for four genomic islands, referred to as *Campylobacter jejuni* integrated elements (CJIEs), and small gene clusters. Recently, a comparative genomic analysis by using DNA microarrays demonstrated that these CJIEs were present in *C. jejuni* strains from various geographical locations and from both clinical and veterinary sources [40], and the coding sequences within the CJIEs from these *C. jejuni* strains were highly divergent when compared to strain RM1221 [40], [44]. Based on these findings, the CJIEs are postulated to be additional hypervariable genomic regions that may contribute to increase the diversity of *C. jejuni* strains [40].

In the present study, a comparative analysis was performed to examine the genetic content of a collection of *C. jejuni* strains recovered from patients admitted to the Red Cross War Memorial Children's Hospital in Cape Town, South Africa. In the Cape Town area, GBS has been estimated to occur at a high incidence in children under 15 years of age [14], [16]. Based on these findings, it has been postulated that a severe form of GBS may be prevalent in this area. Among *C. jejuni* strains from South African patients with GBS, serotype HS∶41 has been found to be overrepresented [14], [16]. In this comparative genomic analysis, sequence-based typing methods, MLST and whole-genome DNA microarrays, were employed to identify distinguishing features within the genomes of South African *C. jejuni* strains with different serotypes that were recovered from patients with GBS, MFS or enteritis.

## Materials and Methods

### Bacterial strains, growth conditions and chemicals

The *C. jejuni* reference and clinical strains used in this study are shown in Table 1. The source and characteristics of the *C. jejuni* strains, isolated from patients admitted to the Red Cross War Memorial Children's Hospital in Cape Town, South Africa, have been described previously [14], [16]. *C. jejuni* strains were grown at 42°C under microaerobic conditions (8% CO_2_, 4% O_2_, 80% N_2_, 8% H_2_) on Oxoid anaerobe basal agar (Remel Inc., Lenexa, KS) amended with 5% laked horse blood (Hema Resource and Supply Inc., Aurora, OR). PCR enzymes and reagents were purchased from New England Biolabs (Beverly, MA) or Epicentre Biotechnologies (Madison, WI). DNA sequencing chemicals and capillaries were purchased from Applied Biosystems (Foster City, CA). All other chemicals were purchased from Sigma-Aldrich Chemicals (St. Louis, MO) or Fisher Scientific (Pittsburgh, PA).

**Table 1:** MLST analysis of the *Campylobacter jejuni* strains used in this study.

Strains^a^	Year	Country^c^	Disease^d^	LOS class	Penner type(s)^e^	ST	Allele number						
							*aspA*	*atpA*	*glnA*	*gltA*	*glyA*	*pgm*	*tkt*
RM1221 (ATCC BAA-1062)	1996	USA	Unk	F	HS∶53	354	8	6	10	2	2	11	12
RM1862 (NCTC 11168)	Unk^b^	UK	Enteritis	C	HS∶02	43	2	5	1	5	3	4	1
RM3148 (ATCC BAA-530)	Unk	MEX	GBS	A	HS∶41	1672	1	8	2	42	4	256	9
RM3149 (ATCC BAA-529)	Unk	MEX	GBS	A	HS∶41	1672	1	8	2	42	4	256	9
RM3193 (260.94)	1994	SA	GBS	A	HS∶41	362	1	8	2	49	4	11	66
RM3194 (285.94)	1994	SA	Enteritis	B	Unk	1471	24	6	171	2	2	89	59
RM3196 (233.94)	1994	SA	GBS	A	HS∶41	362	1	8	2	49	4	11	66
RM3197 (308.95)	1995	SA	GBS	A	HS∶41	362	1	8	2	49	4	11	66
RM3198 (367.95)	1995	SA	GBS	A	HS∶41	362	1	8	2	49	4	11	66
RM3201 (378.96)	1996	SA	Enteritis	A	HS∶41	362	1	8	2	49	4	11	66
RM3203 (16.97)	1997	SA	Enteritis	H	HS∶12	137	4	1	7	10	4	42	7
RM3204 (20.97)	1997	SA	Enteritis	H	HS∶12	137	4	1	7	10	4	42	7
RM3205 (199.97)	1997	SA	Enteritis	A	HS∶41	362	1	8	2	49	4	11	66
RM3206 (242.98)	1998	SA	MFS	A	HS∶41	362	1	8	2	49	4	11	66
RM3207 (250.97)	1997	SA	Enteritis	A	HS∶41	362	1	8	2	49	4	11	66
RM3208 (1.98)	1998	SA	Enteritis	H	HS∶21	137	4	1	7	10	4	42	7
RM3209 (24.98)	1998	SA	Enteritis	H	HS∶12	137	4	1	7	10	4	42	7
RM3211 (96.00)	2000	SA	GBS	A	HS∶33	1472	2	127	2	42	4	90	25
RM3430	Unk	CAN	Unk	A	HS∶41	41	16	8	2	16	62	3	9
RM4186 (390.96)	1996	SA	Enteritis	Unk	Unk	354	8	6	10	2	2	11	12
RM4187 (172.03)	2003	SA	Enteritis	F	Unk	1473	28	129	34	27	33	45	36
RM4191 (160.03)	2003	SA	Enteritis	G	Unk	587	1	8	2	42	4	90	25
RM4192 (124.03)	2003	SA	Enteritis	F	Unk	1474	14	6	17	5	2	2	3
RM4193 (MF 321317.03)	2003	SA	MFS	B	Unk	730	2	5	4	5	2	2	1
RM4194 (180.03)	2003	SA	Enteritis	H	Unk	436	7	44	21	5	62	4	61
RM4196 (126.01)	2001	SA	Enteritis	B	Unk	429	7	5	4	1	2	11	1
RM4197 (MF 996.00)	2000	SA	MFS	F	Unk	257	9	6	2	4	62	4	5
RM4269 (367.95)	1995	SA	GBS	A	HS∶41	362	1	8	2	49	4	11	66
RM4270 (287.96)	1996	SA	Enteritis	A	HS∶41	362	1	8	2	49	4	11	66
RM4271 (302.96)	1996	SA	Enteritis	B	HS∶33	1043	10	7	27	33	44	10	5
RM4273 (375.96)	1996	SA	Enteritis	B	HS∶02	824	9	6	2	2	2	11	5
RM4274 (379.96)	1996	SA	Enteritis	Unk	HS∶42	1475	2	6	4	5	93	11	201
RM4275 (398.96)	1996	SA	Enteritis	H	HS∶37	51	7	12	17	2	15	23	3
RM4277 (422.96)	1996	SA	Enteritis	F	HS∶04,16, 43,50	52	9	6	25	2	10	22	3
RM4278 (423.96)	1996	SA	Enteritis	F	HS∶05	52	9	6	25	2	10	22	3
RM4279 (424.96)	1996	SA	Enteritis	H	HS∶37	51	7	12	17	2	15	23	3
RM4281 (1.97)	1996	SA	Enteritis	C	Unk	19	2	5	1	5	3	2	1
RM4282 (18.97)	1996	SA	Enteritis	H	HS∶50, 62	51	7	12	17	2	15	23	3

a Original strain number is designated in parenthesis.

b Unk, unknown.

c UK, United Kingdom; USA, United States of America; SA, South Africa; MEX, Mexico; CAN, Canada.

d GBS, Guillain-Barré syndrome; MFS, Miller Fisher syndrome.

e Penner heat-stable (HS) serotypes.

### Multilocus sequence typing of *C. jejuni* strains

The *C. jejuni* strains in Table 1 were typed by using the *C. jejuni* MLST primer sets as described previously [34], [45]. The Perl program MLSTparser [45] was used to extract allele sequences and assign allele numbers and sequence types. All allelic sequences were queried against the *Campylobacter jejuni/coli* MLST database (http://pubmlst.org/campylobacter/) to assign numbers to alleles already present in the database. Novel alleles and sequence types (STs) were submitted to the MLST database to obtain new numbers. Each novel *C. jejuni* ST was compared to existing *C. jejuni* STs by concatenating the allelic sequences at all loci for that ST and performing either pairwise BLASTN comparisons against similarly concatenated *C. jejuni* ST sequences or performing CLUSTALX alignments [45], [46]. A dendrogram was constructed by using the aligned concatenated sequences and the neighbor-joining method with the Kimura two-parameter distance estimation method. Subsequent phylogenetic analyses were performed by using MEGA version 2.1, as in previous studies [46].

### Construction of the *C. jejuni* DNA microarray

DNA fragments of individual open reading frames (ORFs) were amplified by using the Sigma-Genosys (The Woodlands, TX) *C. jejuni* ORFmer primer set specific for strain NTCT11168 coding sequences and by using primers from Operon Technologies (Alameda, CA) specific for unique sequences in strain RM1221, as previously described [40]. Additional DNA fragments were amplified from strain RM3193 (Table 1) based on the published sequence for the serotype HS∶41 capsular locus ORFs [35] and for LOS genes from locus classes A, B, C, E and F, as in previous studies [27]. A total of 1530, 227, 40 and 28 PCR products were amplified from strain NCTC 11168, strain RM1221, LOS genes and serotype HS∶41 capsule genes, respectively. The PCR products were purified on a Qiagen 8000 robot by using a Qiaquick 96-well Biorobot kit (Qiagen, Valencia, CA) and spotted in duplicate onto Ultra-GAPS glass slides (Corning Inc., Corning, N. Y.) by using an OmniGrid Accent (GeneMachines, Ann Arbor, MI), as described previously [40]. Immediately after printing, the microarrays were UV crosslinked at 300 mJoules by using a Stratalinker UV Crosslinker 1800 (Stratagene, La Jolla, CA) and stored in a desiccator. Before use, microarrays were blocked with Pronto! Pre-Hybridization Solution (Corning Inc.), according to the manufacturer's specifications.

### Preparation and fluorescent labeling of genomic DNA and microarray hybridization

Genomic DNA from *C. jejuni* was prepared as described previously [27] or purified by using the Wizard Genomic DNA kit (Promega, Madison, WI), according to the manufacturer's specifications. Each microarray hybridization reaction consisted of genomic DNAs from the reference strains (an equal amount of DNA from *C. jejuni* strain NCTC11168 and strain RM1221) and from a test strain that were fluorescently labeled with indodicarbocyanine (Cy5) and indocarbocyanine (Cy3), respectively. When examining the Penner serotype HS∶41 strains, genomic DNA was purified from the reference strain RM3193. Approximately 2 µg of DNA was mixed with 5 µl 10× NEBlot labeling buffer containing random octadeoxyribonucleotides (New England Biolabs, Beverly, MA) and water to a final volume of 41 µl. This mixture was heated to 95°C for 5 min, cooled for 5 min at 4°C and added to the remainder of the labeling reaction consisting of 5 µl of 10× dNTP labeling mix (1.2 mM each dATP, dGTP, dCTP; 0.5 mM dTTP in 10 mM Tris pH 8.0; 1 mM EDTA) (Promega Corporation, Madison, WI), 3 µl of 25 nmole Amersham CyDye fluorescent nucleotides, Cy3-dUTP or Cy5-dUTP (GE Healthcare Life Sciences Corp., Piscataway, NJ), and 5 U of Klenow fragment (New England Biolabs). The labeling reactions were incubated overnight at 37°C, as previously described [40]. Labeled DNA was purified from unincorporated CyDye fluorescent label by using Qiaquick PCR Cleanup kit (Qiagen, Valencia, CA) and dried by vacuum.

### DNA microarray hybridization and data analysis

Labeled reference and test DNAs were combined in a 45 µl Pronto! cDNA hybridization solution (Corning Inc., Corning, NY), heated to 95°C for 5 min and immediately centrifuged at 14,500× *g* for 2 min at room temperature. Fifteen µl of this hybridization mixture was added to each microarray and sealed with a coverslip. The microarray slide was placed in a hybridization chamber (Corning Inc.) and incubated at 42°C for 18 h. Following hybridization, the slides were washed and dried by centrifugation at 300× *g* for 10 min before scanning, as previously described [40]. At least two hybridization reactions were performed for each test strain and were quantified further as described below.

DNA microarrays were scanned by using an Axon GenePix 4000B microarray laser scanner (Molecular Devices Corporation, Sunnyvale, CA) at 532 nm (Cy3) and 635 nm (Cy5) excitation wavelengths with a 10 µm resolution, as described previously [40]. Briefly, after scanning of the DNA microarrays, features and local background intensities were detected and quantified with GenePix 4.0 software (Molecular Devices). Spots were excluded from further analysis if they contained an anomalous spot morphology or were within regions of non-specific fluorescence. The data were filtered by discarding spots with a reference signal lower than the background plus 3 standard deviations of the background. Signal intensities were corrected by subtracting the local background, and calculating the Cy5/Cy3 ratios. To compensate for unequal dye incorporation, data normalization was performed as described previously [40], [47]. Only half of the reference DNA (Cy5-labeled mixture of NCTC 11168 and RM1221 DNA) hybridized to the NCTC 11168 and RM1221 strain-specific spots, increasing the Cy3/Cy5 ratio by twofold. Therefore, the ratios for these NCTC 11168 and RM1221 strain-specific spots were divided by 2 before determining the status of the gene. The ratios for spots of each individual gene were then averaged. As previously described [40], the comparative genomic indexing analysis defined the status of a gene as present when the Cy3/Cy5 (test/reference) intensity ratio was >0.6, as divergent when the Cy3/Cy5 intensity ratio was between 0.6 and 0.3, and as highly divergent or absent when the Cy3/Cy5 intensity ratio was <0.3. For LOS class A, B, and E specific genes that are not present in the reference strains (NCTC 11168 and RM1221), the status of these genes was still determined by the Cy3/Cy5 intensity ratio. Given that the Cy5 intensity represented only nonspecific hybridization, the status of these LOS class-specific genes was defined as present when the Cy3/Cy5 (test/reference) intensity ratio was >3, and as absent when the Cy3/Cy5 intensity ratio was <3. The LOS gene status was confirmed by PCR described previously (Parker, *et al.*, 2005). The status for all genes was converted into trinary scores (present = 2; divergent = 1; highly divergent or absent = 0). The trinary scores for all genes for each strain were further analyzed with GeneSpring microarray analysis software version 7.3 (Agilent Technologies, Santa Clara, CA) and subjected to average-linkage hierarchical clustering with the standard correlation and bootstrapping. The comparative genomic indexing analysis to assign present or absent genes of the GBS-associated strains with HS∶41 serotype was performed by using strain RM3193 as a reference strain and the GENCOM software [41], [48]. For each of these hybridizations, the Cy5 and Cy3 signal intensities were corrected by subtracting the local background before submission into the GENCOM program for determining the present or absent assignments for each gene. The microarray data has been deposited in the NCBI Gene Expression Omnibus (GEO) repository (http://www.ncbi.nlm.nih.gov/geo/) with the accession number GSE9862.

## Results

### Multilocus sequence typing analysis of the South African strains

**Figure 1:**
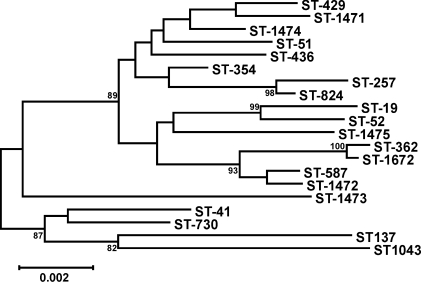
Dendrogram of *Campylobacter jejuni* sequence types, including clinical strains from South Africa, Mexico and Canada. The dendrogram was constructed by using the neighbor-joining algorithm and the Kimura two-parameter distance estimation method. Bootstrap values of >75%, generated from 500 replicates, are shown at the nodes. The scale bar represents substitutions per site.

To examine the genetic variation among 32 *C. jejuni* clinical strains from South Africa, multilocus sequence typing (MLST) was employed to analyze the nucleic acid sequences of seven *C. jejuni* housekeeping loci (*aspA*, *atpA* (*uncA*), *glnA*, *gltA*, *glyA*, *pgm*, and *tkt*). As shown in Table 1, a total of 18 different sequence types (ST) were identified among these *C. jejuni* clinical strains. ST-51, ST-52, ST-137 and ST-362 were represented by more than one strain, and several strains with ST-51, ST-52 and ST-137 had different serotypes (Table 1). In contrast, the analysis demonstrated that *C. jejuni* strains from South Africa with serotype HS∶41 were all assigned ST-362. However, different sequence types were observed for three serotype HS∶41 strains from other geographical locations, Mexico and Canada. Of particular interest, the strains from Mexico shared 4 alleles (*aspA*, *atpA*, *glnA* and *glyA*) with the South African HS∶41 strains. To further examine the relatedness of the serotype HS∶41 strains from these two geographical locations, a dendrogram was constructed by using the neighbor joining method for pairwise comparisons of the concatenated seven allele sequences (Figure 1). The phylogenetic analysis demonstrated that the South African HS∶41 strains, members of ST-362, belong to the same clade as the two ST-1672 strains from Mexico with a bootstrap value of 100% (Figure 1), indicating that these strains from different geographical locations are highly similar by MLST.

### Comparative genomic indexing of the South African strains

**Figure 2:**
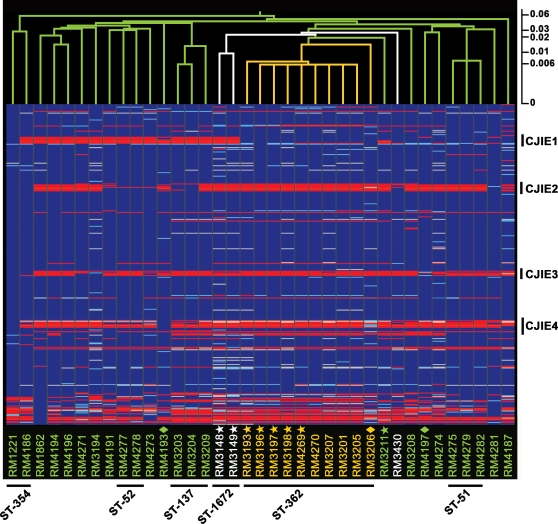
Genome comparison of *Campylobacter jejuni* clinical strains by DNA microarrays analysis. An average linkage hierarchical clustering of the *C. jejuni* strains with a distance score scale bar was compiled in GeneSpring version 7.3 with the standard correlation and bootstrapping (see Materials and Methods). The gene status based on cutoff values of absence and presence predictions is shown color-coded: blue, present; light blue, divergent; red, highly divergent or absent; white, no data. *C. jejuni* strains from South Africa with HS∶41 serotype (yellow), with other serotypes (green), or strains with HS∶41 serotype from Mexico and Canada (white) are designated vertically across the bottom. GBS-associated strains are annotated with stars; MFS-associated strains are annotated with diamonds. The four *C. jejuni*-integrated elements (CJIEs) and the assigned MLST sequence type (ST) for each strain cluster is indicated.

The intraspecies genetic diversity found in the South African clinical strains was examined at the whole-genome level by DNA microarrays. A comparative analysis was performed with a multi-strain *C. jejuni* DNA microarray based on sequence data from the genome strains NCTC 11168 and RM1221 (see Materials and Methods). In this microarray analysis, a hierarchical clustering was performed to further examine the relationship among the clinical strains by using a standard correlation function where the linkage distance between strains is represented by branch lengths in the resulting cluster. Analysis of the comparative microarray data demonstrated that strains with the same sequence type clustered together (Figure 2), demonstrating a strong correlation with our MLST analysis. A distinct subcluster with a distance score of 0.016 was observed for all serotype HS∶41 strains from South African patients with GBS, MFS or enteritis in ST-362 (Figure 2). Clustering next to this group with a distance score of 0.026 were the two GBS-associated HS∶41 strains from Mexico both with ST-1672. In contrast, strains from enteritis cases with distinct serotypes HS∶12 and HS∶37 in ST-137 and ST-51, respectively, formed two separate clusters that showed divergence from the main cluster composed of serotype HS∶41 strains (Figure 2).

**Figure 3:**
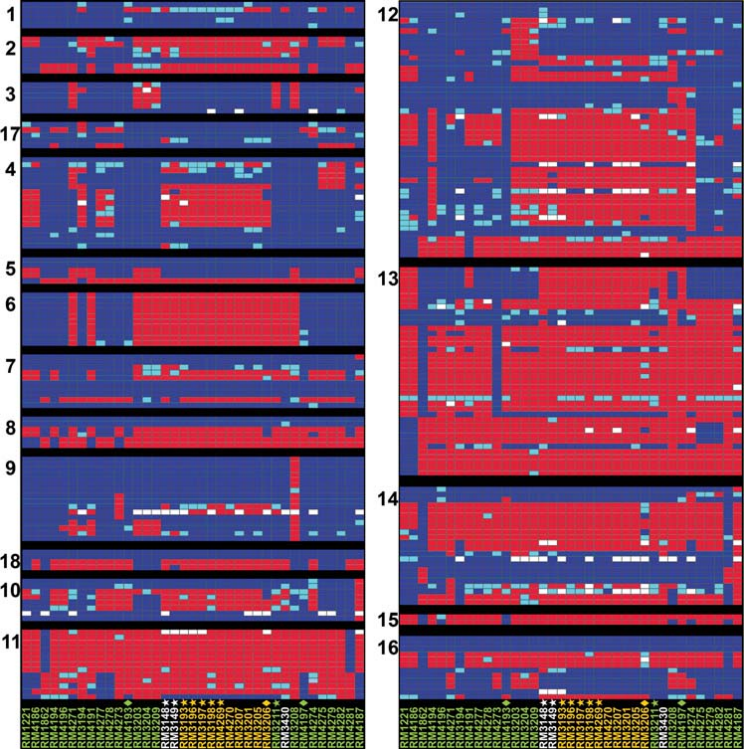
Patterns of presence, absence or divergence in the 18 hypervariable regions in *Campylobacter jejuni* strains. A detailed genomic analysis of the 18 hypervariable regions (Table 2) was compiled in GeneSpring version 7.3 with the standard correlation and bootstrapping (see Materials and Methods). Each panel represents a hypervariable region, and each column corresponds to a *C. jejuni* strain designated vertically across the bottom, as described in the legend to Figure 2. The gene status based on cutoff values of absence and presence predictions is shown color-coded: blue, present; light blue, divergent; red, highly divergent or absent; white, no data.

Using the trinary score for present, absent or divergent genes, 17.8% (314 of 1757) of the genes were absent or highly divergent in at least one South African strain when compared to the genome strains represented on the microarray (Figure 3). Most of the variable genes in the South African strains were found in the hypervariable genomic regions (Table 2), regions previously described to be involved in capsule (region 13) and LOS (region 11) biosynthesis, flagellar modification and O-linked glycosylation (region 12) and type I restriction-modification (region 14) [42]. Further analysis of a subset of the South African strains with the HS∶41 serotype demonstrated that genes in four hypervariable genomic regions (regions 4, 9, 12 and 13) were different from both strains NCTC 11168 and RM1221 (Figure 3). Interestingly, region 4 (CJE0340-CJE0355), encoding proteins involved in pantothenate and biotin biosynthesis and for a molybdenum ABC transporter (Table 2), was the only region that was highly divergent in all HS∶41 strains from South Africa and Mexico but present in the HS∶41 strain from Canada (Figure 3).

**Table 2:** Intraspecies hypervariable regions in *Campylobacter jejuni*.

Region	Genes^a^	Proposed function
1	CJE0031-CJE0035 (Cj0032-Cj0036)	Type IIS restriction/modification
2	CJE0051-CJE0055 (Cj0055c-Cj0059c)	Unknown
3	CJE0170-CJE0175 (Cj0177-Cj0182)	Putative iron transport, biopolymer transport, putative TonB transport protein
17	CJE0308-CJE0313 (Cj0258-Cj0263)	Putative zinc transport; dihydroorotase; homodimeric type
4	CJE0340–CJE0355 (Cj0294-Cj0310c)	Pantothenate and biotin biosynthesis pathway; molybdenum ABC transporter
5	CJE0470-CJE0472 (Cj0421c-Cj0425)	Unknown
6	CJE0530-CJE0538 (Cj0480c-Cj0490)	Unknown; UxaA family hydrolase
7	CJE0666-CJE0673 (Cj0561c-Cj0571)	Unknown
8	CJE0728-CJE0732 (Cj0625-Cj0629)	Hydrogenase formation proteins; type III restriction/modification
9	CJE0828-CJE0844 (Cj0727-Cj0755)	Phosphate regulated genes; iron uptake; TonB transport protein; ferric enterobactin uptake receptor
18	CJE0944-CJE0947 (Cj0857c-Cj0860)	Unknown
10	CJE1047-CJE1056 (Cj0967-Cj0975)	Unknown
11	CJE1278-CJE1281 (Cj1135-Cj1145c)	Lipooligosaccharide biosynthesis
12	CJE1485-CJE1532 (Cj1293-Cj1343)	Flagellar modification; O-linked glycosylation
13	CJE1601-CJE1622 (Cj1414c-Cj1449c)	Capsule biosynthesis
14	CJE1714-CJE1733 (Cj1543c-Cj1563c)	Type I restriction/modification; unknown
15	(Cj1677-Cj1679)	Unknown
16	CJE1888-CJE1896 (Cj1717c-Cj1729c)	2-isopropylmalate synthase; isopropylmalate dehydrogenase; 3-isopropylmalate dehydratase; unknown

a The start and end of each region is shown for genes in strain RM1221 (strain NCTC 11168) [40], [42].

Analysis of the comparative microarray data of the LOS region showed that all six strains from South African patients with GBS possessed class A (Table 1). This finding correlated with previous reports that demonstrated that the class A locus, a class that has been associated with the GM1 ganglioside-like structure [8], [9], [49], was overrepresented in GBS-associated strains [8], [26], [27]. In the present study, this LOS locus class was identified also in the four enteritis-associated strains with the HS∶41 serotype (Table 1). Similar to previous findings [50], most GBS-associated strains examined in this study bound cholera toxin (data not shown), which binds highly selectively to GM1 gangliosides [9], [51], demonstrating that these strains may have a similar LOS outer core structure. In contrast, the GBS-associated strains RM3196 and RM3198 failed to bind cholera toxin. These observations suggested that strains RM3196 and RM3198 with a class A LOS locus may prevalently synthesize ganglioside mimics with an outer core structure that is different from a GM1-like ganglioside, as recently demonstrated [9]. Interestingly, the MFS-associated strains RM3206, RM4193, and RM4197 with distinct LOS locus classes A, B, and F, respectively, also failed to bind cholera toxin (data not shown), as demonstrated previously in a recent study showing a lack of cholera toxin binding by all MFS-associated strains [50].

To identify potential differences within the genome of GBS-associated strains, the genetic relatedness of the HS∶41 South African strains from GBS and enteritis patients was examined further by using the GENCOM software [41], [48] and the GBS-associated strain RM3193 as a reference strain (see Materials and Methods). In this particular analysis, the cutoff values between divergent and similar genes were established to represent a 95% nucleotide sequence identity for each gene examined between the reference strain RM3193 and the remaining South African strains with HS∶41 serotype (Table 1). The regression slope had a linear relationship between the logarithms of the red Cy5 (reference RM3193 strain) and the green Cy3 (tested HS∶41 strain) fluorescence (data not shown). These results indicated that the genome content of these GBS- and enteritis-associated strains with HS∶41 serotype, a serotype considered to be clonal and highly stable [19], was very similar. Based on this DNA microarray analysis, differences in gene content could not be identified in the HS∶41 South African strains from GBS and enteritis patients.

**Figure 4:**
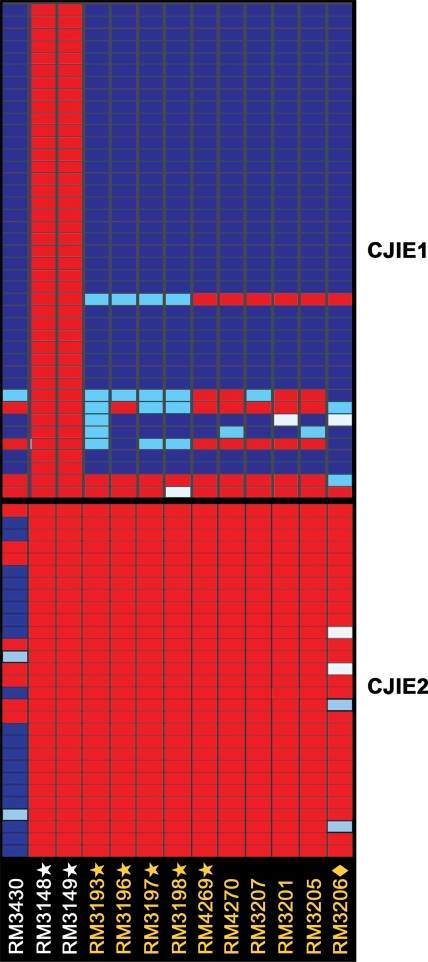
Patterns of presence, absence or divergence in the *Campylobacter jejuni*-integrated elements. A detailed genomic analysis of the genomic integrated elements CJIE1 (top) and CJIE2 (bottom) was compiled in GeneSpring version 7.3 with the standard correlation and bootstrapping (see Materials and Methods). Each column corresponds to a *C. jejuni* strain designated vertically across the bottom, as described in the legend to Figure 2. The gene status based on cutoff values of absence and presence predictions is shown color-coded: blue, present; light blue, divergent; red, highly divergent or absent; white, no data.

The comparative genomic analysis further assessed for the presence of CJIEs, genomic integrated elements initially identified in strain RM1221 [40], [43], in the *C. jejuni* strains from South Africa. From the hierarchical cluster analysis that examined the relationships among the four CJIEs for each strain, the presence of genes in at least one of the four CJIEs was identified in each South African strain (Figure 2). Interestingly, the clustering analysis demonstrated that all serotype HS∶41 strains from South Africa contained most genes in the CJIE1 genomic element, as in strain RM1221 (Figure 4), whereas genes in the other three genomic elements, CJIE2, CJIE3, and CJIE4, were absent in all of these strains (Figures 2 and 4). By using our trinary score for genes present, absent or divergent in sequence (see Materials and Methods), no distinct features in the CJIE1 element were observed for any of the HS∶41 South African strains from GBS patients when compared to strains from enteritis patients (Figure 4). Furthermore, the hybridization patterns for the genes in the CJIEs were compared with serotype HS∶41 strains from other geographical locations. The hierarchical analysis demonstrated the presence of genes for both CJIE1 and CJIE2 genomic elements in strain RM3430, a serotype HS∶41 strain from Canada (Figure 4). When compared to CJIE genes represented in the DNA microarray, both CJIE1 and CJIE2 genes were absent in the Mexican HS∶41 strains RM3148 and RM3149 (Figure 4). These results indicated that the inclusion of CJIE sequences, RM1221-like genomic elements, in our microarray analysis distinguished the gene content of the clonal HS∶41 South African strains from three HS∶41 strains from Canada and Mexico.

## Discussion

The present study performed a comparative genomic analysis, by using the sequence-based typing methods MLST and DNA microarrays, to examine the genetic polymorphism of *C. jejuni* strains that were isolated from South African patients with various disease outcomes, enteritis, GBS or MFS. The hierarchical cluster analysis by DNA microarrays showed that *C. jejuni* strains with the same sequence type clustered together. Our MLST and microarray analysis also demonstrated that all of the South African strains with HS∶41 serotype, a serotype considered to be clonal and highly stable [19], are clearly distinct from the other South African strains and that they are also distinct from other HS∶41 strains from other three strains from Canada and Mexico. Although several reports demonstrated regions of high variability between other strains of *C. jejuni* and strain NCTC 11168 [36]–[39], [41], [42], the gene indexing analysis performed in this study, by using a microarray containing genes from both genome strains NCTC11168 and RM1221 [40], identified a total of 18 intraspecies hypervariable regions in the genome from the South African strains, as previously reported for other *C. jejuni* strains from various geographical locations and sources [40]. Intriguingly, only hypervariable region 4 (CJE0340-CJE0355), encoding proteins involved in pantothenate and biotin biosynthesis and for a molybdenum ABC transporter, was highly divergent in all HS∶41 strains from South Africa and Mexico but present in the HS∶41 strain from Canada. A previous study documented that this region was distinct between strain NCTC 11168 and RM1221, and similar results were obtained when the hybridization pattern for additional *C. jejuni* isolates from various locations and sources was examined [40]. These findings have led to the proposal that an additional level of diversity may be found in the hypervariable region 4 [40]. Still to be determined is the role of genomic diversity within this region and whether the divergence of these genes is characteristic of certain serotype HS∶41 strains.

Inclusion in this microarray of the coding sequences within the four CJIEs, identified initially in strain RM1221 [40], [43], differentiated the South African HS∶41 strains from the two closely-related HS∶41 strains from Mexico. Only CJIE1, a *Campylobacter* Mu-like prophage [40], [43], was present in the South African HS∶41 strains whereas all four CJIEs were absent in the HS∶41 strains from Mexico. A more distantly-related HS∶41 strain from Canada possessed both CJIE1 and CJIE2. In addition, the presence of bacteriophage-related genes in CJIE1 may probably contribute to increasing the genomic diversity of these *C. jejuni* strains. These findings demonstrated that these CJIEs are additional regions of high variability between strains of *C. jejuni* and that these genomic regions could be utilized in the differentiation of closely-related *C. jejuni* strains.

The comparative analysis further investigated any potential association between specific classes of LOS biosynthesis loci and GBS. Our DNA microarray analysis demonstrated that all of GBS-associated strains possessed the class A LOS whereas only 17% (4 of 24) of the enteritis-control strains also possessed this class of LOS. Indeed, it has been speculated that the *C. jejuni*-induced neuropathies are probably related to the molecular mimicry between gangliosides in nerve tissue and LOS on the *C. jejuni* cell surface [7]. Biochemical and serological studies have revealed that GBS-associated strains exhibit ganglioside-like structures in their LOS [50], [52]. More specifically, strains possessing the class A locus have been shown to express an LOS outer core that mimics gangliosides [23]–[25], and the occurrence of the class A locus has been correlated strongly with GBS-associated strains [8], [9], [26], [27]. Finally, the only substantial differences between GBS-associated and control *C. jejuni* strains appears to date to reside in the LOS-biosynthetic gene locus [8], [25], [26], [53]. Recent comparative genomic analysis have proposed that GBS markers may be located only in the LOS biosynthesis region [25], [53], [54].

Previous studies that employed MLST and DNA microarrays could not identify GBS- specific genetic markers by comparing the genomes of *C. jejuni* strains from various geographical locations, serotypes, and disease outcomes [39], [55]. Furthermore, no molecular markers specific to the GBS syndrome were detected after analyzing a highly clonal HS∶41 population from South African patients by using a different typing technique, high-throughput amplified fragment length polymorphism [53]. Similar results were obtained in the present study by using DNA microarrays to compare the genome content of South African *C. jejuni* GBS-associated and enteritis strains with the HS∶41 serotype. There are several potential reasons why no GBS-specific molecular markers in *C. jejuni* were identified in this study. First, our DNA microarray was based on the genome sequence of strains NCTC 11168 and RM1221, and genes that are not present in these strains used to construct the microarray will not be detected. Furthermore, single nucleotide changes that may result in point mutations leading to biological differences, but not detectable by DNA microarrays, may be an important factor in the development of GBS. However, these genetic differences could be disclosed potentially by a more detailed DNA analysis identifying single-nucleotide polymorphisms (SNPs). Just recently, SNPs have been used successfully for comparing entire genomes in clinically-relevant strains of *Mycobacterium tuberculosis* [56], *Helicobacter pylori* [57], *E. coli* [58] and *S. enterica* [59]. Furthermore, specific *C. jejuni* GBS-features may rather involve differential expression of *Campylobacter* virulence factors and could be revealed by expression analysis of GBS-associated strains. Indeed, this approach might identify regulatory or virulence-related genes or gene loci that represent mechanisms important in the development of molecular mimicry associated with GBS. Given that there is broad variability in the severity and spectrum of clinical symptoms in GBS patients [12], [13], host-susceptibility may play a substantial role in the development of GBS [60], [61]. Thus, further characterization of the interaction between the host and *C. jejuni* is needed to better understand the pathogenesis of *Campylobacter*-induced GBS.
